# Invasive coronary physiology in patients with angina and non-obstructive coronary artery disease: a consensus document from the coronary microvascular dysfunction workstream of the British Heart Foundation/National Institute for Health Research Partnership

**DOI:** 10.1136/heartjnl-2021-320718

**Published:** 2022-03-22

**Authors:** Divaka Perera, Colin Berry, Stephen P Hoole, Aish Sinha, Haseeb Rahman, Paul D Morris, Rajesh K Kharbanda, Ricardo Petraco, Keith Channon, UK Coronary Microvascular Dysfunction Working Group, Divaka Perera

**Affiliations:** 1 British Heart Foundation Centre of Excellence and National Institute for Health Research Biomedical Research Centre, School of Cardiovascular Medicine and Sciences, King’s College London, London, UK; 2 BHF Glasgow Cardiovascular Research Centre, University of Glasgow, Glasgow, UK; 3 Cardiology, Golden Jubilee National Hospital, Clydebank, UK; 4 NIHR Cambridge Biomedical Research Centre, Cambridge, UK; 5 Infection, Immunity and Cardiovascular Disease, The University of Sheffield, Sheffield, UK; 6 Department of Cardiovascular Medicine, University of Oxford, Oxford, UK; 7 National Heart and Lung Institute, Imperial College London, London, UK

**Keywords:** Coronary Angiography, Angina Pectoris, Chest Pain, Microvascular Angina

## Abstract

Nearly half of all patients with angina have non-obstructive coronary artery disease (ANOCA); this is an umbrella term comprising heterogeneous vascular disorders, each with disparate pathophysiology and prognosis. Approximately two-thirds of patients with ANOCA have coronary microvascular disease (CMD). CMD can be secondary to architectural changes within the microcirculation or secondary to vasomotor dysfunction. An inability of the coronary vasculature to augment blood flow in response to heightened myocardial demand is defined as an impaired coronary flow reserve (CFR), which can be measured non-invasively, using imaging, or invasively during cardiac catheterisation. Impaired CFR is associated with myocardial ischaemia and adverse cardiovascular outcomes.

The CMD workstream is part of the cardiovascular partnership between the British Heart Foundation and The National Institute for Health Research in the UK and comprises specialist cardiac centres with expertise in coronary physiology assessment. This document outlines the two main modalities (thermodilution and Doppler techniques) for estimation of coronary flow, vasomotor testing using acetylcholine, and outlines a standard operating procedure that could be considered for adoption by national networks. Accurate and timely disease characterisation of patients with ANOCA will enable clinicians to tailor therapy according to their patients’ coronary physiology. This has been shown to improve patients’ quality of life and may lead to improved cardiovascular outcomes in the long term.

## Introduction

Stable ischaemic heart disease comprises a broad range of coronary pathophysiology associated with a spectrum of prognostic outcomes and potential therapies.^1^ The aim of diagnostic testing is to elucidate whether there is an ischaemic mechanism causing anginal chest symptoms and to determine the optimal therapies for individual patients. We now know that a large proportion of patients with angina, or indeed demonstrable myocardial ischaemia, have non-obstructed coronary arteries (ANOCA or INOCA).^2 3^ ANOCA is an umbrella term, which comprises different pathophysiological disease entities. These include coronary microvascular disease (CMD), coronary endothelial dysfunction and epicardial coronary vasospasm, each with distinct prognostic outlooks and guideline-directed therapies.^4^ The presence of coronary vascular abnormalities corresponds with myocardial ischaemia and abnormal coronary perfusion during exercise,^5^ as well as greater morbidity and mortality, and represents a modifiable therapeutic target, which should therefore be proactively identified.^6 7^ Over the past decade, there has been a rapid growth in the evidence base supporting routine physiological assessment to guide management of patients with ANOCA, with a recent strengthening to IIA recommendation in the current ESC guidelines.^1^ In patients who are referred for angiography and found to have functionally non-obstructive disease, the catheter laboratory visit represents an ideal opportunity to resolve diagnostic ambiguity, improve patient outcomes and optimise resource utilisation.

The UK CMD workstream is an initiative supported by the NIHR-BHF Cardiovascular Partnership, which aims to standardise and harmonise coronary vascular physiology assessment in the UK enabling research involving data collected during standard care and in clinical research studies. The partnership aims to promote clinical research collaborations through international networks. This document sets out the core assessments that need to be performed to identify the presence of coronary vascular abnormalities in patients presenting to the cardiac catheterisation laboratories.

## Coronary vascular physiology assessment in patients with ANOCA

Coronary vascular assessment can be performed readily and safely and provides accurate and reproducible evaluation of microvascular function.^8^ Fractional flow reserve (FFR) quantifies the functional significance of epicardial coronary artery stenoses, but does not allow additional physiological assessment of patients with ANOCA. The main parameter used to distinguish normal from abnormal coronary microvascular function is the coronary flow reserve (CFR). CFR is the ratio of hyperaemic to baseline coronary blood flow (CBF) and reflects the ability to augment myocardial blood supply in response to increased demand, determined by the extent to which microvascular resistance can be dynamically decreased. CMD can be secondary to architectural changes within the microcirculation, such as vascular smooth muscle hypertrophy and capillary rarefaction, or vasomotor dysfunction, such as endothelial and/or vascular smooth muscle dysfunction. An inability to adequately augment CBF is characteristic of CMD and is associated with increased likelihood of myocardial ischaemia and adverse cardiovascular outcomes.^6 9^ While absolute CBF (mL/min) is difficult to measure in a clinical setting, it can currently be estimated by one of the two techniques: *Doppler* to measure coronary flow velocity or *Thermodilution* to measure the mean transit time of room temperature saline, each requiring the use of different sensor-tipped, ultra-thin, intracoronary (IC) guidewires. CBF is estimated at rest and in response to pharmacological stressors, like adenosine (to test endothelium-independent function) and acetylcholine (ACh; to test endothelium-mediated vasodilatation).

In the absence of obstructive epicardial coronary artery disease (CAD), an impaired CFR (defined as <2.5) confirms the diagnosis of endothelium-independent CMD, while an impaired acetylcholine flow reserve (AChFR) (defined as ≤1.5) confirms the diagnosis of coronary endothelial dysfunction.^10 11^ The Coronary Vasomotion Disorders International Study Group (COVADIS) states that CMD can be diagnosed at a CFR threshold of 2.0 or 2.5.^12^ A recent study has demonstrated that patients with CFR 2.0–2.5 are physiologically indistinguishable from those with CFR <2.0.^10^ This study found the optimal dichotomous CFR threshold for predicting global myocardial ischaemia to be 2.5 (sensitivity 95%, specificity 65%; area under the curve=0.80, p<0.001). Importantly, myocardial perfusion and exercise physiology parameters of patients with CFR 2.0–2.5 resembled those of patients with CFR <2.0. Therefore, CFR <2.5 is an accurate surrogate of (substrate for) myocardial ischaemia, with a high sensitivity and reasonable specificity.

The combined use of adenosine and ACh provides the highest accuracy when confirming or excluding the presence of an ischaemic substrate in patients with ANOCA.^10^ This has significant implications on patient management and resource utilisation. The CorMicA study has demonstrated that stratifying treatment in patients with ANOCA based on coronary vascular physiology assessment yields superior outcomes to empirical therapy, supporting the role of coronary physiology testing in this patient cohort.^3 8^

## Patient selection

Patient selection is critical to ensure optimal resource utilisation and to offer maximal benefit to the appropriate patient cohort. For these reasons, invasive coronary vascular physiology assessment should be reserved for patients with a high pre-test probability of coronary vascular dysfunction, i.e. patients with cardiovascular risk factors, typical symptoms of angina despite optimal medical therapy ±evidence of ischaemia on stress imaging, in the absence of obstructive epicardial CAD.

## Protocol

Patients with symptoms suggestive of myocardial ischaemia who are referred for coronary angiography and found to have unobstructed coronary arteries (confirmed with pressure-based indices as appropriate, namely FFR>0.80 or non-hyperaemic pressure ratio >0.89) should proceed to estimation of coronary flow by using thermodilution or Doppler-based techniques. CFR calculated by the two techniques are both reliable in identifying myocardial ischaemia validated against positron emission tomography imaging.^13^

### Vascular access

We recommend using the radial artery as the preferred route of access, given the overwhelming safety data associated with this strategy. Intra-arterial nitrates may be used to prevent the occurrence of radial artery spasm; these have a short half-life and are unlikely to affect the integrity of results obtained.

### Target coronary artery

We recommend using the left anterior descending (LAD) artery as the target coronary artery for assessment. The LAD subtends a large percentage of the myocardium, and much of the evidence base on CFR in ANOCA is derived from measurements made in the LAD. If the LAD artery cannot be used for technical reasons, then we recommend using the left circumflex artery, followed by the right coronary artery (RCA). Operators may also wish to assess a specific coronary artery if there is objective evidence of localised ischaemia or vasospasm elsewhere.

To avoid catheter or wire thrombosis, intravenous or intra-arterial heparin (70–100 U/kg) should be administered to achieve therapeutic anticoagulation (activated clotting time 250 s) before coronary instrumentation.

### Doppler-based technique (ComboWire, Philips Volcano, California)

Following intubation of the target vessel, the ComboWire (fitted with pressure and Doppler sensors) is advanced until the pressure sensor is between the guide catheter and the ostium of the coronary artery in question. The introducer needle should be withdrawn, the catheter flushed with saline and the ComboWire pressure equalised to the catheter signal. The ComboWire should then be manipulated into the mid-to-distal LAD (at least 5 cm from the ostium) and fine rotational movements applied to obtain optimal and stable Doppler traces using the density of the signal on the visual display of the console as well as the phasic auditory signal. Optimal readings occur when the Doppler probe is aligned co-axially with vessel. When using a wire with offset sensors, it is desirable to manipulate the wire so that the tip is in a retroflex (or looped) orientation, which allows a more stable signal and ensures that the pressure and Doppler sensors are in the same location within the artery.

Once an optimal Doppler signal is obtained, the catheter laboratory team should optimise the signal to noise ratio by varying the instantaneous peak velocity threshold and/or the display threshold. On most consoles, the flow velocity scales will be set to auto adjust, although manual setting of the velocity scale can allow further optimisation (especially in instances of 50 Hz electrical interference) in the hands of experienced teams.

#### Endothelium-independent function assessment (CFR)

IC nitrate should be administered before any readings are taken. We suggest using lower doses of short-acting glyceryl trinitrate (GTN) (≤200 µg). The half-life of GTN is approximately 2 min, which makes it an appealing choice in case ACh assessment is planned later. The short half-life of GTN should prevent false negative outcomes during the subsequent ACh assessment. Once steady state is achieved, the baseline coronary pressure and ﬂow measurements can be taken.

**Figure 1:**
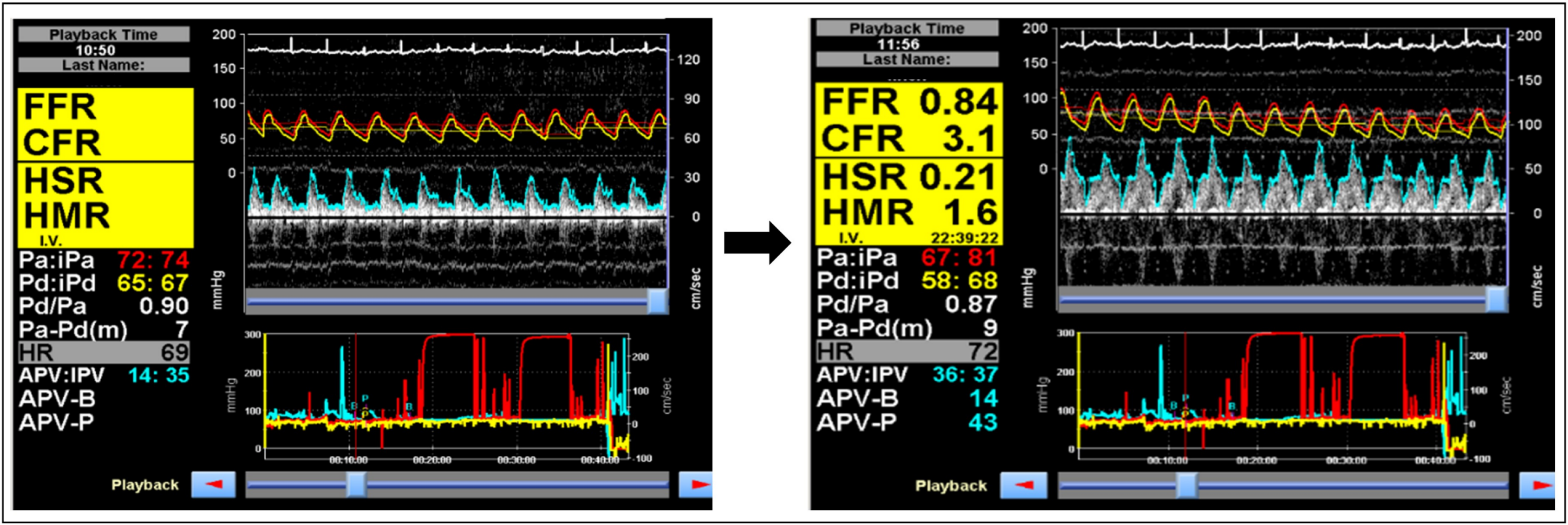
Coronary vascular physiology assessment, using Doppler wire, demonstrating normal epicardial coronary artery (FFR=0.84) and endothelium-independent coronary microvascular (CFR=3.1) physiology. The aortic (red) and distal coronary (yellow) pressures are used to calculate Pd/Pa and FFR. The Doppler flow signals are used to derive the instantaneous peak velocity (IPV) and averaged peak velocity (APV), which are used as a surrogate for coronary blood flow. The pressure and flow traces are both ECG-gated. CFR=APV-P/APV-B; hMR=Pd/APV-P; hSR=(Pa-Pd)/APV. APV-B, basal averaged peak velocity; APV-P, hyperaemic averaged peak velocity; CFR, coronary flow reserve; FFR, fractional flow reserve; HMR, hyperaemic microvascular resistance; HSR, hyperaemic stenosis resistance.

Once stable pressure and flow signals are achieved, the baseline average peak velocity (APV) should be documented. Hyperaemia should now be induced either by intravenous administration of adenosine at a dose of 140 µg/kg/min or by IC bolus of adenosine (use maximum dose tolerated in relation to the occurrence of atrioventricular block, usually 60–120 µg in the left coronary artery (LCA) and 30–60 µg in the RCA). Hyperaemia is confirmed when the APV increases, as the microvascular resistance drops significantly, and the steady-state APV is taken to be the hyperaemic APV. Other markers of hyperaemia include systemic symptoms related to IV adenosine, ventricularisation of the distal coronary pressure waveform, disappearance of the distal dicrotic pressure notch and separation of mean aortic and distal pressures. The CFR should be calculated once maximal hyperaemia is confirmed, and good quality flow signals are achieved by adjusting scales and tracking (figure 1). CFR <2.5 is suggestive of CMD.

The formula used by the console to calculate CFR is as follows:

CFR=APV_hyper_/APV_rest_.

(APV=average peak velocity; hyper=hyperaemia; rest=resting conditions)

#### Coronary endothelial function assessment (AChFR)

Coronary endothelial function is assessed using ACh infusion. At the time of writing, in the UK, ACh is usually supplied as powdered Miochol eyedrops. Twenty milligrams of powdered Miochol is diluted into 500 mL sterile normal saline (0.9%), forming 40 µg/mL stock solution; 22.5 mL of this stock solution is drawn up into a 50 mL Luer Lock syringe and made up to 50 mL with normal saline (ACh solution concentration 18.2 µg/mL).

Defibrillator pads and ECG leads should be applied prior to ACh testing and the team made aware that external pacing may become necessary, although this is a rare complication. Prior to administration of ACh, the ComboWire should be positioned in the target artery and the flow signal optimised. IC ACh, at concentrations of up to 10^−4^ mol/L (to achieve estimated final blood concentrations in the coronary bed of up to 10^−6^ mol/L), is infused over 2 min. The exact regimen may differ according to local procedures or clinical considerations.^14^ Two examples of infusion regimes currently used in the UK are as follows: an infusion of three sequential concentrations, 0.182, 1.82 and 18.2 µg/mL at 1 mL/min for 2 min infusion periods; an infusion of 18.2 µg/mL at 1 mL/min for 2 min followed by 2 mL/min for 2 min. It should be noted that these rates apply to infusion into the LCA; the infusion rate or dose should be halved for the RCA.

Since the ACh is infused directly via the guide catheter, continuous aortic pressure recordings are interrupted during this period. Measurement of distal coronary pressure and flow from the ComboWire can give an indication of coronary spasm, in addition ECG changes and patients’ symptoms should be closely monitored. An alternative approach would be to position an infusion catheter (eg, using a coronary microcatheter) in the mid vessel and infuse the ACh solution continuously through this (with the guide catheter in the coronary ostium). If this approach is adopted, then the ComboWire should be placed just distal to the tip of the infusion catheter. This technique allows for continuous aortic pressure to be monitored throughout the ACh infusion protocol.

At the end of the ACh infusion, a syringe should be connected to the guide catheter and used to aspirate the ACh remaining in the catheter. This avoids accidently delivering a bolus of ACh if the catheter is flushed with saline or contrast.

Pre-ACh and post-ACh cine images should be obtained for quantitative coronary angiography (QCA). The images should be from two angles at least 30° apart and assessed in the view with the least foreshortening of the artery.

The estimation of volumetric flow from Doppler flow velocity also incorporates vessel diameter. Given that ACh can cause either epicardial vasodilation or vasoconstriction, volumetric CBF should always be calculated (whether vasoconstriction is obvious or not), using QCA to estimate epicardial diameter.

Coronary blood flow=0.5 × π(average peak velocity) (vessel diameter/2)^2^.

Vessel diameter is calculated 5 mm distal to the Doppler wire.

AChFR is calculated as CBF_ACh_ /CBF_rest_.

An AChFR of ≤1.5 is suggestive of coronary endothelial dysfunction^11^ and is associated with adverse cardiovascular outcomes.^7^ As with CFR, AChFR lies on a continuous spectrum with a lower value conferring higher risk.

The procedure of using ACh to test endothelial function in the cardiac catheter laboratory has been found to be safe and well tolerated by patients.^15^ Given ACh’s short half-life and the relatively low concentrations used in the regime above, adverse events are rare. In the unlikely event that patients develop atrioventricular block, reducing the rate of, or stopping, the infusion should mitigate this situation, with IV atropine and transcutaneous pacing as backup measures. If patients develop epicardial vasospasm, then operators should inject IC nitrates, which should quickly mitigate the spasm. In the unlikely event that nitrates do not ameliorate the coronary vasospasm, operators may use IC atropine to reverse the effects of ACh.

### Bolus thermodilution technique (PressureWire X, Abbott Vascular, Santa Clara, California)

PressureWire X (fitted with temperature and pressure sensors) and the Coroventis software (Coroventis AB, Uppsala, Sweden) are used for the thermodilution technique. Following normal pressure wire set up procedures, the PressureWire X is connected wirelessly and auto-calibrated outside the body. Following intubation of the target vessel with a suitable guide catheter, the wire can be advanced until the pressure sensor (at the proximal end of the radiopaque section, 30 mm from the tip) is at the tip of the guide catheter. After flushing the catheter with saline, the wire pressure is equalised with the aortic pressure at the ostium of the guide catheter. Once it is confirmed that these pressures are equal, the wire is advanced into the distal target artery (at least 5 cm from the catheter ostium). After confirming that the pressure trace from the wire is stable, with no whip artefact or damping, this position should then be maintained throughout the study. It is important to ensure the guiding catheter is engaged, but not so deeply as to ‘plug’ into the left mainstem, especially during hyperaemia, because this will confound the physiological results.

#### Endothelium-independent function assessment (CFR)

IC nitrate should be administered before any readings are taken. Once steady state is achieved, the baseline coronary pressure and ﬂow measurements can be taken.

Thermodilution should be performed using a 5 mL syringe to ensure rapid manual IC injection of 3–4 mL of sterile, room temperature normal saline via the guide catheter (which should be adequately engaged in the coronary ostium). The diagnostic guidewire position in the target coronary artery should be maintained. The mean transit time (Tmn) should be measured on the Coroventis console. The Tmn measurement should be repeated, with a consistent injection volume and force each time, until three readings within ±10% of each other are achieved. The average of these three recordings should be used as the resting flow Tmn value.

Once a resting Tmn measurement has been recorded, hyperaemia should be induced by intravenous administration of adenosine at a dose of 140 µg/kg/min. Hyperaemia should be confirmed by markers described in the Doppler section. At maximal, steady-state, coronary microcirculatory hyperaemia, thermodilution should be repeated as above until three Tmn readings within ±10% of each other are achieved. The average of these three recordings should be used as the hyperaemic flow Tmn value.^16^

**Figure 2:**
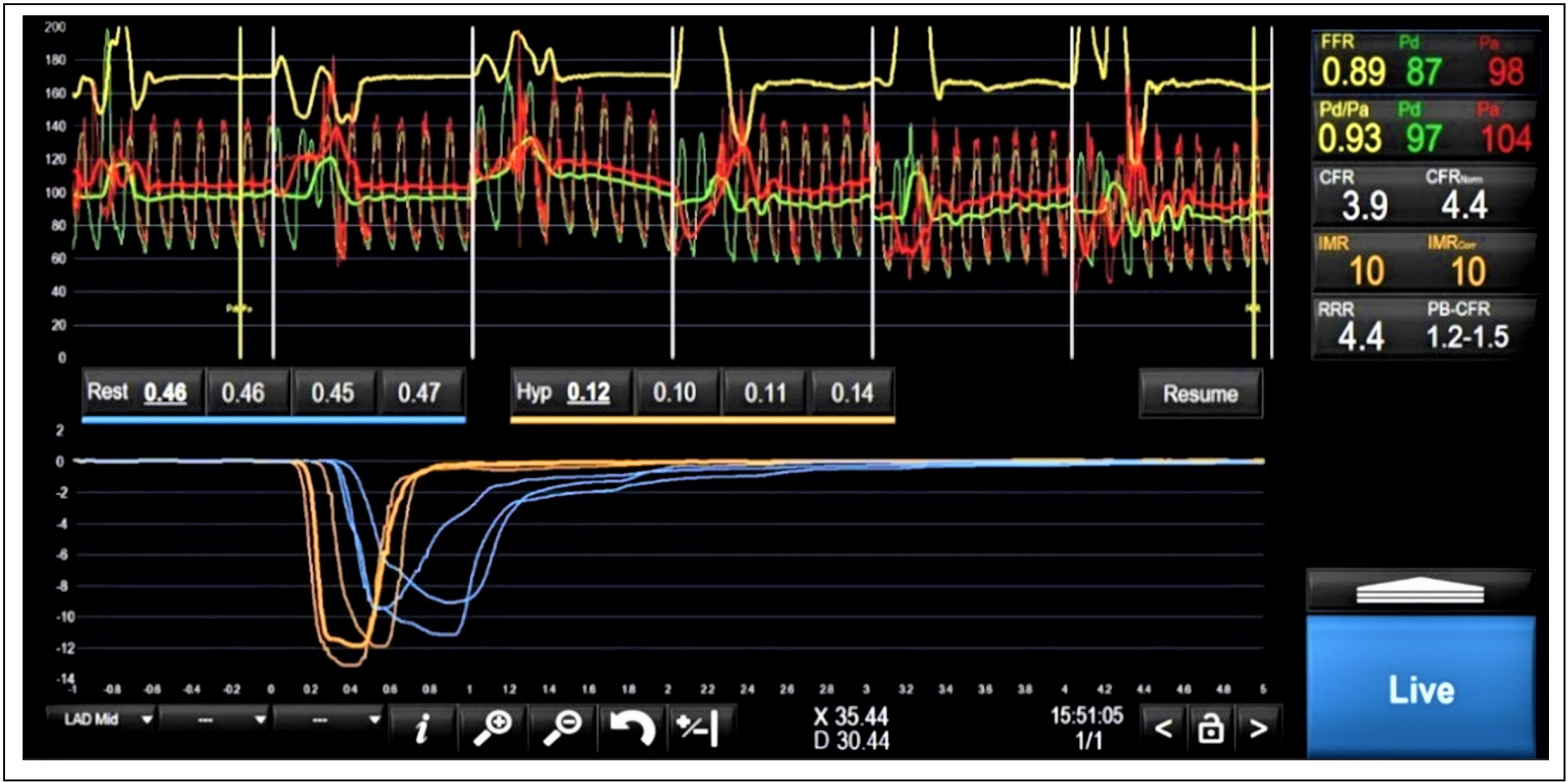
Coronary vascular physiology assessment demonstrating normal epicardial coronary artery (FFR=0.89) and endothelium-independent coronary microvascular (CFR=3.9) physiology. The red (aortic) and green (distal coronary) pressure traces at the top half of the figure are used to calculate Pd/Pa and FFR. The blue traces at the bottom of the figure represent resting transit times (0.46 s, 0.45 s and 0.47 s) and the yellow traces represent hyperaemic transit times (0.10 s, 0.11 s and 0.14 s). CFR=resting mean transit time/hyperaemic mean transit time; IMR=Pd * hyperaemic mean transit time. CFR, coronary flow reserve; FFR, fractional flow reserve.

Once resting and hyperaemic measurements are completed, the CFR should be displayed on the Coroventis system (figure 2). CFR <2.5 is suggestive of CMD.

The formulae used by the console to calculate CFR is as follows:

$$CFR=\frac{hyperaemic\;flow}{baseline\;flow}=\frac{\left(\frac{1}{{Tmn}_{hyper}}\right)}{\left(\frac{1}{{\text{Tmn}}_{rest}}\right)}=\frac{{Tmn}_{rest}}{{Tmn}_{hyper}}$$

The pressure wire should now be withdrawn to the coronary ostium to check for drift on the pressure signal of the wire relative to the guiding catheter. If there is ≥0.03 drift, then the operator should re-equalise and repeat the measurements.

### Continuous thermodilution technique (PressureWire X, Abbott Vascular)

Continuous thermodilution, to measure absolute flow (Q) and resistance (R), has now become commercially available. The measurements are safe and feasible^17^ and have been validated against positron emission tomography.^18^ The setup requires a PressureWire X to be placed in the distal vessel; a monorail infusion catheter is then placed in the proximal vessel over the wire. Continuous thermodilution measurements are performed by infusing room temperature IC saline, via the infusion catheter, at a set rate while the temperature in the distal vessel is recorded by the temperature sensor on the PressureWire X. After a steady period of recording, the temperature sensor is then pulled back to the opening of the infusion catheter and the infusion temperature is determined. The saline infusion is then stopped. Using established thermodilution equations, absolute flow (Q) and resistance (R) can be calculated from this dataset.

### Angiographic assessment of coronary vasospasm

**Figure 3:**
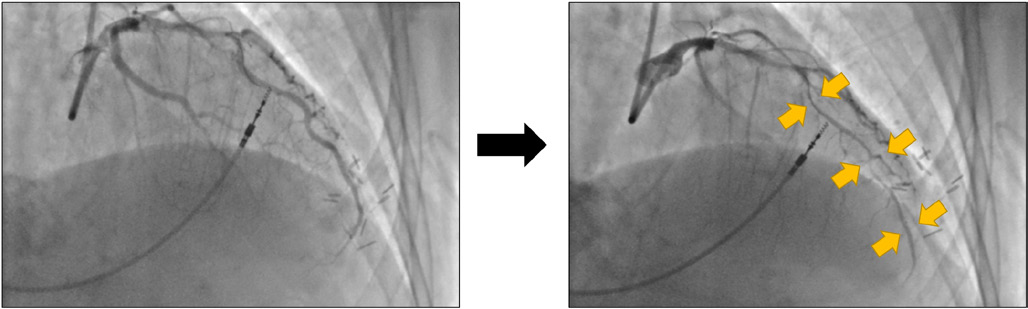
Coronary angiography images of the left coronary artery at baseline (left hand side) and after acetylcholine bolus (right hand side), in a patient with epicardial coronary artery vasospasm. The gold arrows demonstrate the diffuse segments of spasm in the left anterior descending artery.

In cases where the pre-test probability of coronary vasospasm is high, such as in patients with combination of exertional and resting symptoms,^19^ operators may carry out coronary vasospasm provocation assessment using ACh bolus. 5.5 ml of the 18.2 µg/mL ACh solution, 5.5 mL should be withdrawn into a syringe and injected into the LCA over 20 s (delivering a dose of 100 µg over 20 s); this can be performed by hand or using a variable rate infusion pump. If the LCA cannot be used, then the operator should inject 2.75 mL of the 18.2 µg/mL ACh solution into the RCA over 20 s (delivering a dose of 50 µg over 20 s). Operators should take two orthogonal angiographic images before and after the ACh bolus to identify epicardial coronary artery vasoconstriction (figure 3). A diagnosis of vasospastic angina should be made when patients have ≥90% coronary vasoconstriction, ischaemic ECG changes and chest pain.^20^

When using angiographic measures alone the diagnosis of microvascular spasm (by COVADIS criteria) is one of exclusion, namely the occurrence of ischaemic ECG changes and chest pain in the absence of epicardial vasoconstriction. However, where continuous measures of flow are available (for instance, by Doppler flow velocity), the diagnosis of microvascular spasm can be based on the physiological responses. In particular, in situations where there is no change in epicardial artery diameter, isolated microvascular spasm causes a decrease in flow compared with the resting state, and hence can be reliably diagnosed by demonstration of an AChFR <1.0. Given that alterations in CBF occur earlier in the ischaemic cascade than ECG changes and chest pain, the continuous measurement of coronary flow velocity provides a more sensitive diagnostic test of coronary microvascular spasm.

### Alternative vasomotor testing protocols

While our protocol is a comprehensive assessment of the coronary vasculature, alternative protocols exist in other centres, and although the underlying scientific rationale and vasoactive agents remain the same, there can be differences in the doses of agents being used. These protocols deviate most when it comes to assessing patients’ response to ACh. The Women’s Ischaemia Syndrome Evaluation group and Mayo clinic group protocols, for example, assess coronary endothelial function by infusing incremental doses of ACh, at 0.182, 1.82 and 18.2 µg/mL over 3 min.^2 15^ On the contrary, the Academic Medical Centre^21^ and Japanese Cardiology Society^22^ protocols provoke coronary vasospasm using incremental infusion of ACh, at 0.86, 8.63, 86.3, 863 µg/mL over 3 min or 100–200 µg boluses of ACh over 20 s, respectively. The lack of uniformity in coronary endothelial function and/or vasospasm assessment has important clinical implications. For example, when assessing for coronary vasospasm, a 200 µg bolus is more likely to lead to multivessel spasm than a 100 µg bolus.^23^ Additionally, a 20 s bolus dose is more likely to lead to vasospasm compared with a 3 min infusion of the same dose.^24^ This may be because it is the peak blood concentration of ACh, rather than simply the peak dose of ACh, which determines the arterial response to ACh provocation. Furthermore, there remains uncertainty surrounding the optimal dose and infusion rate of ACh that leads to physiological degrees of spasm that is a surrogate for what patients experience during day-to-day life; the caveat being that beyond a certain threshold of dose and infusion rate ACh may provoke spasm in any individual, as evidenced by 97% prevalence of vasospasm with ACh provocation in a recent prospective registry.^25^ Additionally, there may be gender-specific differences in responses to incremental doses of ACh.^26^

In view of the protocol disparities described, we advocate for an international consensus, whereupon the regimen and doses of vasoactive agents utilised are standardised. This is not only important for diagnosis and overall patient care but also to ensure homogeneity when recruiting patients into therapeutic trials.

**Figure 4:**
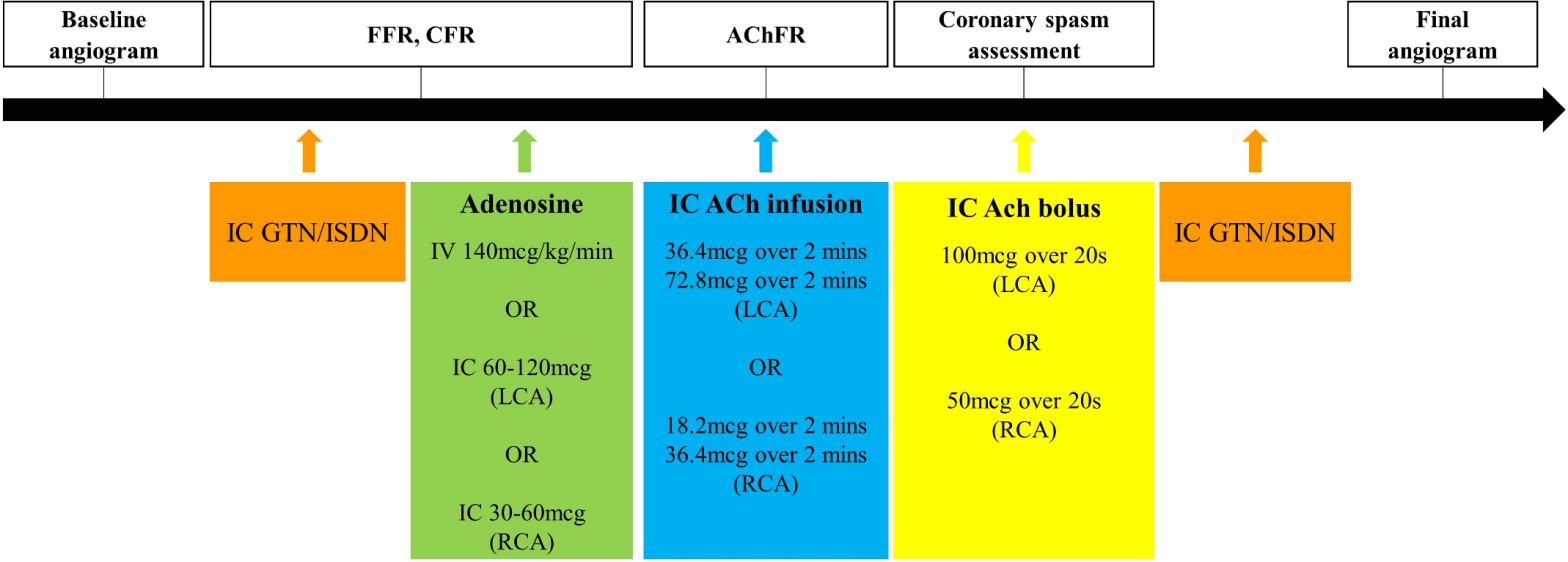
The sequence of vasodilator infusions utilised to assess coronary vascular physiology in the catheter laboratory. AChFR, acetylcholine flow reserve; CFR, coronary flow reserve; FFR, fractional flow reserve; GTN, glyceryl trinitrate; IC, intracoronary; ISDN, isosorbide dinitrate; LCA, left coronary artery; RCA, right coronary artery.

**Figure 5:**
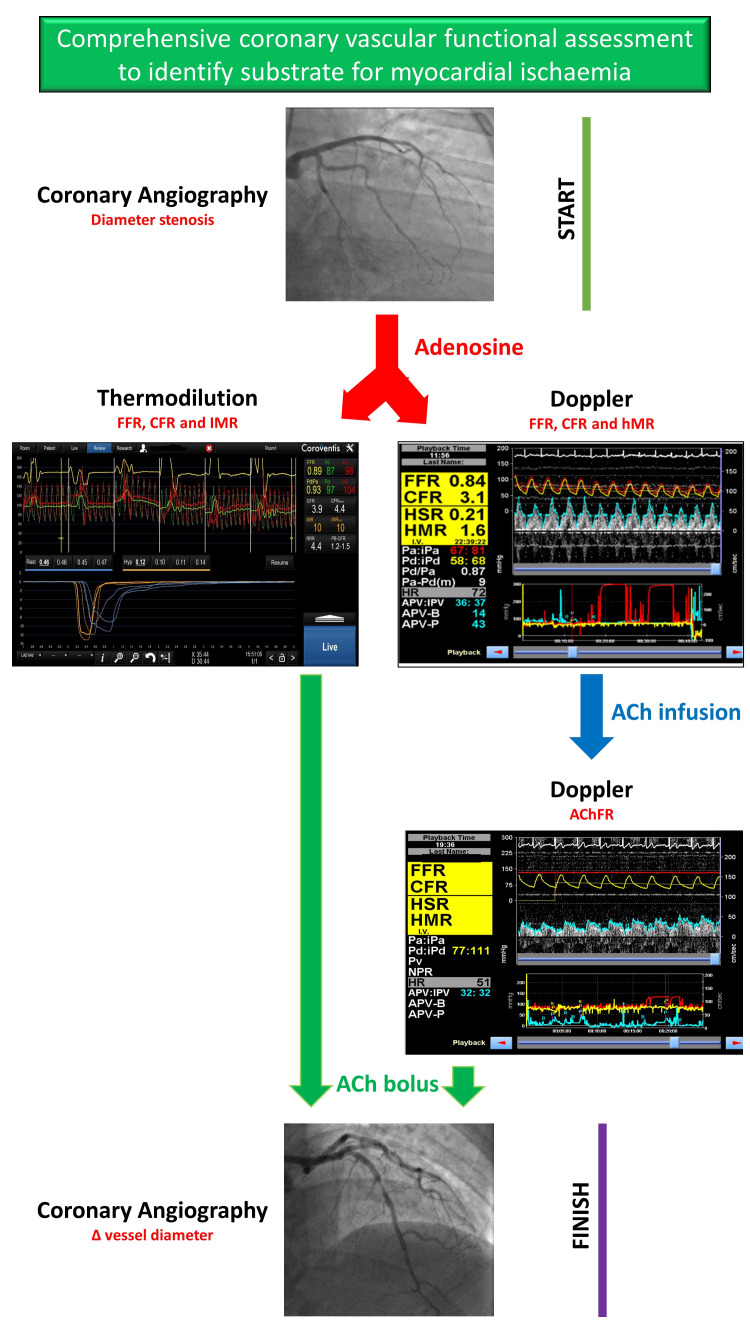
This figure represents our proposed algorithm for any patient coming to the catheter laboratory with symptoms suggestive of myocardial ischaemia who is found to have unobstructed epicardial arteries. AchFR, acetylcholine flow reserve; CFR, coronary flow reserve; FFR, fractional flow reserve; hMR, hyperaemic microvascular resistance; HSR, hyperaemic stenosis resistance.

Figure 4 illustrates the sequence of vasodilators and the appropriate measurements made during coronary vascular physiology assessment, whilst figure 5 represents our proposed algorithm for any patient coming to the catheter laboratory with symptoms suggestive of myocardial ischaemia who is found to have unobstructed epicardial arteries.

## Standardised reporting

**Figure 6:**
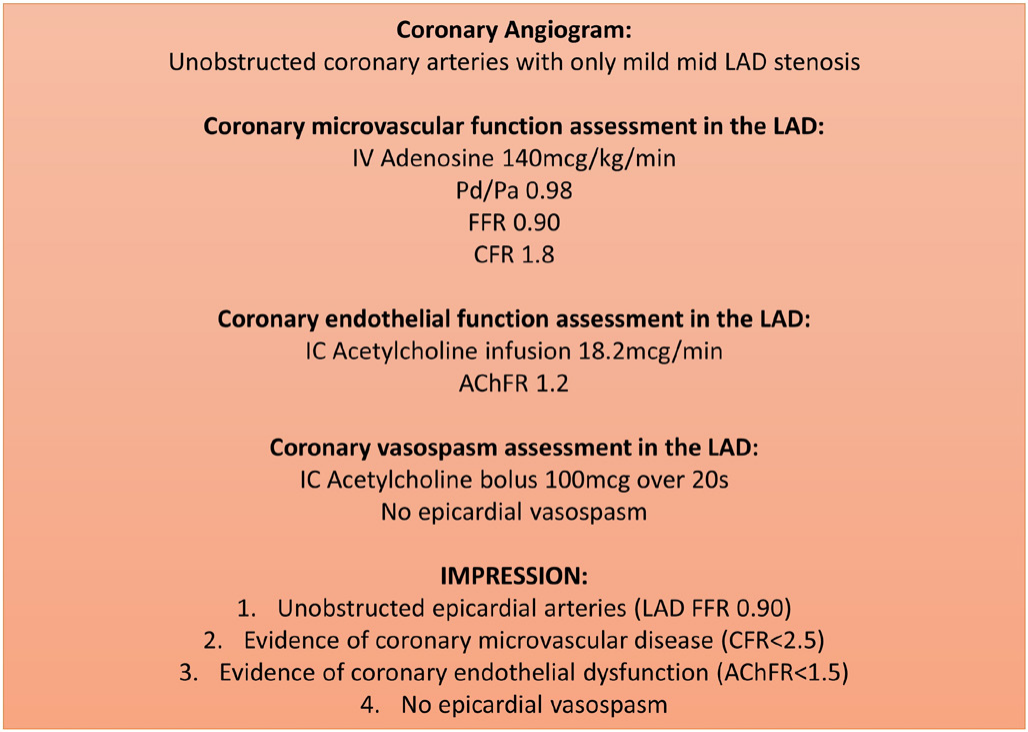
An example of a standardised report for a patient with coronary vascular physiology assessment. We recommend recording the following data on every report: (1) epicardial artery indices (FFR/NHPR); (2) coronary endothelium-independent microvascular function (CFR); (3) coronary endothelial function (AChFR) (if measured) and (4) evidence of coronary vasospasm (if measured), including maximum induced diameter stenosis, symptoms and ECG changes; (5) systemic haemodynamics: aortic blood pressure, left ventricular end-diastolic pressure and rate pressure product. AChFR, acetylcholine flow reserve; CFR, coronary flow reserve; FFR, fractional flow reserve; LAD, left anterior descending.

Following a comprehensive assessment of coronary vascular physiology, we recommend standardised reporting to ensure that uniform diagnostic thresholds are being used to derive the final diagnosis as this has direct implications on patients’ management and prognosis. The minimum dataset will depend on the resources available and alignment with the format used for reporting routine cardiac catheterisation procedures within each network. An example of a standardised report is included in figure 6.

## Therapeutic implications

The major driver of poor quality of life and adverse cardiovascular outcomes in patients with ANOCA is the cessation of prognostic and anti-anginal therapy due to false reassurance. Without a better understanding of patients’ coronary vascular physiology, physicians are unable to provide personalised treatment strategies. The BHF CorMicA study has demonstrated the utility of personalised therapy, based on coronary vascular physiology assessment, in improving patients’ quality of life.^3^ The stratified medicine intervention was also associated with a favourable health economic evaluation.^27^ At present, there is a paucity of evidence-based treatment options for patients with CMD and coronary endothelial dysfunction and trials are ongoing to study the effects of known and repurposed therapies on both prognosis and symptom control in patients with CMD.^28^ Online supplemental figure 1 summarises the current anti-anginal options available for patients with coronary artery disease.

10.1136/heartjnl-2021-320718.supp1 Supplementary data

## Conclusion

Coronary vascular physiology assessment allows clinicians to distinguish between the disparate pathologies that cannot be differentiated based on a coronary angiogram alone. This can inform about the prognosis and promote personalised therapy. There is now a strong mandate to assess the coronary vascular physiology in patients with ANOCA. This consensus document provides operators with a practical template of how to conduct a comprehensive coronary vascular physiology assessment, along with discussing the potential pitfalls and complications from carrying out these assessments. An increased uptake of coronary vascular physiology assessment may lead to a better quality of life, prognosis and resource utilisation in patients with ANOCA. However, contemporary evidence linking specific therapies to outcomes is still awaited.
