# Supplementary figures and images for: Invasive coronary physiology in patients with angina and non-obstructive coronary artery disease: a consensus document from the coronary microvascular dysfunction workstream of the British Heart Foundation/National Institute for Health Research Partnership
Source: Heart. 2022 Mar 22;109(2):88–95. doi: 10.1136/heartjnl-2021-320718 (PMC9811089; doi:10.1136/heartjnl-2021-320718)

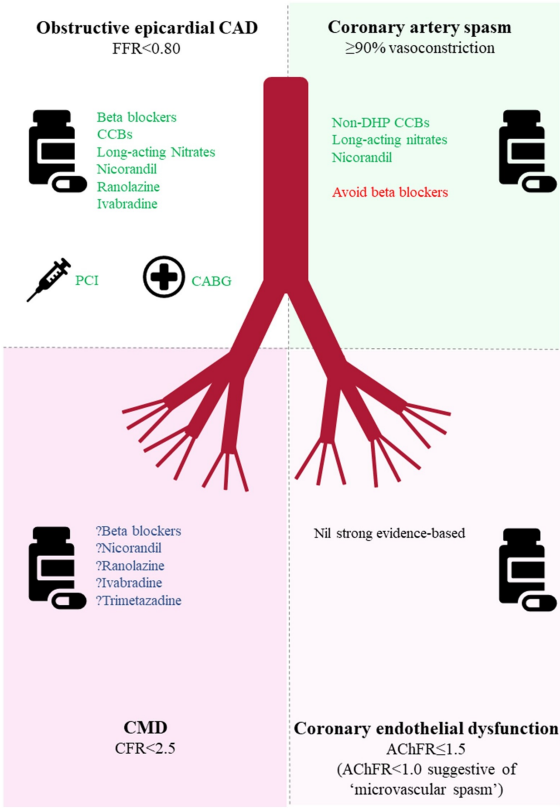

Supplement: Supplementary data [file heartjnl-2021-320718supp001.pdf]
